# Effects of Developmental Bisphenol A Exposure on Reproductive-Related Behaviors in California Mice (*Peromyscus californicus*): A Monogamous Animal Model

**DOI:** 10.1371/journal.pone.0055698

**Published:** 2013-02-06

**Authors:** Scott A. Williams, Eldin Jasarevic, Gregory M. Vandas, Denise A. Warzak, David C. Geary, Mark R. Ellersieck, R. Michael Roberts, Cheryl S. Rosenfeld

**Affiliations:** 1 Bond Life Sciences Center, University of Missouri, Columbia, Missouri, United States of America; 2 Division of Biological Sciences, University of Missouri, Columbia, Missouri, United States of America; 3 Interdisciplinary Neuroscience Program, Center for Translational Neuroscience, University of Missouri, Columbia, Missouri, United States of America; 4 Department of Psychological Sciences, University of Missouri, Columbia, Missouri, United States of America; 5 Thompson Center for Autism and Neurodevelopmental Disorders, University of Missouri, Columbia, Missouri, United States of America; 6 Department of Animal Sciences, University of Missouri, Columbia, Missouri, United States of America; 7 College of Agriculture, Food, and Natural Resources- Statistician, University of Missouri, Columbia, Missouri, United States of America; 8 Department of Biochemistry, University of Missouri, Columbia, Missouri, United States of America; 9 Genetics Area Program, University of Missouri, Columbia, Missouri, United States of America; 10 Department of Biomedical Sciences, University of Missouri, Columbia, Missouri, United States of America; Michigan State University, United States of America

## Abstract

Bisphenol A (BPA), a pervasive, endocrine disrupting compound (EDC), acts as a mixed agonist- antagonist with respect to estrogens and other steroid hormones. We hypothesized that sexually selected traits would be particularly sensitive to EDC. Consistent with this concept, developmental exposure of males from the polygynous deer mouse, *Peromyscus maniculatus*, to BPA resulted in compromised spatial navigational ability and exploratory behaviors, while there was little effect on females. Here, we have examined a related, monogamous species, the California mouse (*Peromyscus californicus*), where we predicted that males would be less sensitive to BPA in terms of navigational and exploratory behaviors, while displaying other traits related to interactions with females and territorial marking that might be vulnerable to disruption. As in the deer mouse experiments, females were fed either a phytoestrogen-free CTL diet through pregnancy and lactation or the same diet supplemented with BPA (50 mg/kg feed weight) or ethinyl estradiol (EE) (0.1 part per billion) to provide a “pure” estrogen control. After weaning, pups were maintained on CTL diet until they had reached sexual maturity, at which time behaviors were evaluated. In addition, territorial marking was assessed in BPA-exposed males housed alone and when a control male was visible in the testing arena. In contrast to deer mice, BPA and EE exposure had no effect on spatial navigational skills in either male or female California mice. While CTL females exhibited greater exploratory behavior than CTL males, BPA exposure abolished this sex difference. BPA-exposed males, however, engaged in less territorial marking when CTL males were present. These studies demonstrate that developmental BPA exposure can disrupt adult behaviors in a sex- and species-dependent manner and are consistent with the hypothesis that sexually selected traits are particularly vulnerable to endocrine disruption and should be a consideration in risk assessment studies.

## Introduction

Bisphenol A (BPA) is an endocrine disrupting compound (EDC) present in a wide assortment of plastic and cardboard items, dental sealants, and other products [1]–[3]. As a weak estrogen, BPA acts predominantly through estrogen receptor-α and –β (ESR1 and 2), but effects operating through other steroid receptors and pathways have been inferred [4]. In general, it acts as an agonist, but there are indications that at some target sites, including the hippocampus, BPA can antagonize the actions of estrogens and androgens [5], [6]. BPA is currently produced in amounts exceeding 8 billion pounds per year and highly pervasive in the environment [7]. In 2012, the Food and Drug Administration (FDA) banned the production of baby bottles and sippy cups containing BPA [8], but, this restriction fails to address exposure of the fetus and infant through other routes of exposure, in particular the mother. There is abundant evidence in humans and rodents that BPA can transit across the maternal to the fetal placenta and, in lactating dams, is incorporated into the milk and transferred to the suckling infant [9]–[13]. Moreover, fetuses and neonates lack many of the enzymes needed to metabolize BPA and may, therefore, experience greater concentrations of active BPA than the dam [10]–[12]. This development period is characterized by programming of the brain by steroid hormones [14]–[16]. Exposure to endocrine disrupting compounds (EDC), such as BPA, target estrogen receptors (ESR) and other steroid receptors, and EDC are hence likely to disrupt the normal steroid-induced neurogenesis and synaptogenesis that accompany normal brain development [6], [17]–[19]. Experiments designed to test whether developmental exposure to BPA and other EDC might disturb later behavioral traits depend on the choice of animal model and its sex, since some traits and some species may be better gauges of developmental exposure than others.

Animal and human studies indicate that BPA exposure is associated with compromised cognitive abilities, altered reproductive and neuro-affective behaviors, and the neural networks that underpin these traits [20]–[24], although such data have not been universally accepted [25], [26], and the suggestion has been made that the animal models used for testing BPA effects might be inappropriate. We have proposed that examining the effects of EDC on behavior might be most productively performed on those behaviors that have evolved through sexual selection, since they are likely to be particularly vulnerable to compounds in the environment that mimic steroid hormones. Sexual selection usually involves intrasexual competition for mates and intersexual choice of mating partners [27], [28]. For example, in the polygynous deer mouse, *P. maniculatus*, scramble competition for females, which is believed to increase selection for home range expansion during the breeding season appears to provide males with greater spatial navigational abilities than females [29]–[33]. This cognitive trait is considered to be sexually selected in that it provides a competitive advantage to those males with the enhanced information processing skills necessary to locate prospective breeding females widely dispersed throughout the environment. In contrast to males, female deer mice do not expand home territory during the breeding season. Such enhanced behavior in the female of the species would have less value and may even place them at greater risk of predation. Enhanced spatial ability in males is dependent on exposure to sex steroids during fetal and early post-natal brain development and, in the adult animal, on a rise in testosterone concentrations as day length increases during the breeding season [34]. We hypothesized that behaviors yielding a reproductive benefit in one sex but not the other would be particularly susceptible to developmental exposure to BPA and other EDC, such as ethinyl estradiol (EE), that, due to their actions through estrogen and other steroid receptor, could potentially interfere with the normal steroid-induced brain programming occurring during early development [35], [36]. Consistent with our predictions, developmental exposure to BPA at environmentally relevant concentrations diminished sex differences in spatial abilities and exploratory behavior, with the decrease in sex differences largely related to poorer spatial navigational skills in BPA-exposed males. In addition, during mate preference testing, females spent less time engaged in nose-to nose contact with BPA-exposed males than they did with CTLs, indicating that they favored unexposed males [35]. On the other hand, while such previous studies are consistent with the hypothesis that sexually selected traits are susceptible to BPA, these studies alone are not definitive.

To test whether or not EDC, such as BPA, disrupt behavior in a sex- and species-dependent manner, we chose a species, *Peromyscus californicus* (California mouse), closely related to *P. maniculatus*, but socially monogamous. We had previously questioned whether or not there were sex differences in spatial learning under long day conditions in *P. californicus* and found that there were not [37], an outcome consistent with the hypothesis that spatial ability is not a sexually selected trait in this species. In contrast to what is observed in the deer mouse, however, male California mice increase their reproductive success by remaining with one female and investing in biparental care, rather than expanding their territory to search for multiple partners [38], [39]. Hence, female choice is an important mechanism of sexual selection in this species. Even though the role of the father in California mice may be primarily care of offspring [38], the shared home ranges between males and females, high levels of paternal investment, and the sexual receptivity of some female California mice to outside males also creates conditions that likely favor the evolution of male mate guarding and territorial defense [40]–[42]. Male territorial marking is thus an attendant sexually selected trait in males California mice, as is spatial navigation is in male deer mice. Our prediction was that California male mice developmentally exposed to BPA would demonstrate reduced territorial marking relative to CTL, unexposed males. By contrasting the effects of BPA in the California mouse with those of the related polygynous deer mouse, inferences might then be drawn about how EDC, such as BPA, disrupts behaviors that vary according to sex and the evolutionary life history of the species.

## Materials and Methods

### Animal Husbandry

Outbred adult California mouse females and males, free of common rodent pathogens, were purchased from the *Peromyscus* Genetic Stock Center (PGSC) at the University of South Carolina (Columbia, SC), and placed in quarantine for a minimum of 8 weeks to ensure that they did not carry any transmittable and zoonotic diseases. From the time the animals had been captured from the 60 founders collected between 1979 and 1987 in the Santa Monica Mountains in California, *P. californicus* captive stocks have been carefully bred by the PGSC to maintain their outbred status. All experiments were approved by University of Missouri Animal Care and Use Committee (Protocol Number 6564) and performed in strict accordance with the recommendations in the Guide for the Care and Use of Laboratory Animals of the National Institutes of Health.

Virgin females, 8 to 12 wks of age, were randomly assigned to receive one of three diets: (**i**) a low phytoestrogen AIN 93G diet supplemented with 7% by wt corn oil (CTL) to minimize potential phytoestrogenic contamination that would otherwise be present with inclusion of soybean oil in the diet (n = 22 dams); (**ii**) AIN93G supplemented with 50 mg of BPA/kg fw (n = 14 dams); (**iii**) AIN93G diet supplemented with 0.1 parts per billion of EE (n = 10 dams), as the FDA required positive CTL for BPA studies [43]. The doses were chosen based on our previous studies [35], [44]. Under our conditions of housing, unpaired female California mice consumed 3.0±0.5 g/day, leading to an exposure dose of ∼0.15 mg BPA/day. Diets were administered 2 weeks prior to mating, and dams remained on the diet throughout pregnancy and lactation, as sexual differentiation of the brain extends into the early postnatal period [15]. Since California mice are monogamous, one male was paired with a single female, and the pair remained together for the duration of the study. California mice typically birth one to two pups in each litter [42], [44], [45]. Therefore, to obtain sufficient sample sizes some of the animals were rebred for up to 3 parities. The sample size of adult offspring/sex/litter used in the Barnes Maze and Elevated Plus Maze testing is CTL = 17 litters, 19 female and 18 male offspring; BPA = 13 litters, 12 female and 15 male offspring; EE = 9 litters, 10 female and 9 male offspring. Animal numbers were comparable to those employed in previous studies with deer mice and California mice [35], [44]. Based on a Power Analysis [46] and the previous studies, the sample sizes were judged to be sufficient to provide definitive outcomes.

After weaning, all offspring were placed on the CTL diet and housed individually until sexual maturity (age **≈**90 d). To minimize background exposure to BPA beyond treatment regimen, deer mice were housed in white polypropylene cages (32 cm x 18 cm x 24 cm) containing Aspen shavings (NEPCO, Warrensburg, NY) under standard conditions (25±2°C and 50% ±10% humidity), with ad libitum access to BPA-free water provided in glass bottles and diet specific to each treatment group. All animals were maintained on a long day light cycle (16 h light:8 h dark) to induce sexual maturity [45]. To reduce any potential social housing and accompanying dominance/subordinate effects [47]–[49], mice were moved into single-housing conditions at weaning (35 days of age).

### Spatial Learning

The Barnes maze was used to test spatial learning and memory, but modified for *Peromyscus* [35], [44]. This dry-land, circular maze was employed to determine whether each animal was able to learn intra-maze or extra-maze spatial cues to escape the platform into a home cage. The animal was motivated to solve the maze by mildly aversive stimuli, including a stimulatory 1200 lux light (versus ∼400 lux for vivarium room lighting), and, in the cases where where the light alone was insufficient, a recording of a barn owl (*Tyto alba*) was played to motivate predator avoidance and thus maze escape [35], [44], [50]. A detailed description of the particular Barnes maze used in this and earlier studies has been described elsewhere [35], [44].

Each animal was assigned an escape-hole number, with hole numbers for consecutively tested mice shifted by 90°, to eliminate odor cues. Animals were provided two habituation trials followed by seven days of two-trial evaluations per day lasting for 300 sec each, with a 30-min inter-trial interval. The trials were recorded and quantified with an EthoVision XT video camera (Noldus Technologies, Leesburg, VA), and latency (time to enter escape-hole), path length, and error rate tracked by using the automated tracking EthoVision XT software. Performance was averaged across trials on the same day for each individual. The search strategy employed was classified as random, serial, or direct [35], [44]. Random search strategy (coded 1) was reflected by the animal repeatedly crossing the center of the maze before locating the correct escape hole. A serial search strategy (coded 2) was delineated as investigation of consecutive holes in an apparently systematic manner involving primarily a clockwise or counterclockwise motion around the perimeter of the maze. Lastly, a direct search strategy (coded 3) was defined as the animal navigating directly to the target quadrant without crossing the center of the maze more than once and demonstrating three or fewer errors before locating the correct hole [51].

### Exploratory and Anxiety-like Behavior

Exploratory and anxiety-like behaviors of the animals were measured 24 h after the Barnes maze assessment by using the elevated plus maze (EPM), as described previously [35], [36], [44], [52]. Each animal was placed on the center of the platform. The 300 sec trials were recorded with EthoVision XT software (Noldus Technologies, Leesburg, VA, USA), which automatically scored total time spent in open and closed arms and number of closed and open arm entries and center entries.

### Territorial Marking Behavior

Territorial marking behavior was assessed by UV examination of urinary marking patterns, as described previously [53]. Briefly, testing was conducted at 2300 h, 1 h after lights out, with 10 (5 CTL, 5 BPA) single-housed, sexually naïve, age-matched, non-littermate males. Additionally, each male was within ∼5 g of weight of each other to account for potential weight-related social dominance. These requirements, of necessity, limited the number of males available for the experiment, and therefore it was not possible to include the EE-exposed males in the experimental design. Once males were matched, they remained in this particular dyad across all days of testing. Basal urinary marks were obtained on “d 0″ by placing each individual mouse for a duration of 1 h in a polypropylene cage used to house rats (17×23×45 cm), which was lined with two sheets of Whatman No. 4 filter paper (Amazon, Seattle, WA) to collect all of the urine samples. Each male was placed in a separate, clean cage to avoid any pheromone cues that may confound baseline marking. Twenty four h later, the pre-determined dyad was placed on opposite sides of a large cage that was divided by a wire-mesh barrier with each side lined by a single piece of Whatman No. 4 paper. This approach allowed the males to be visible to each other and exchange pheromone cues but not physically interact. After d 1, the animals were co-housed for one hour daily for five consecutive days without a barrier to permit physical interaction between the males. On d 7, marking behavior was analyzed for each pair by once again placing them on opposite sides of a wire-mesh barrier lined on each side by filter paper for 60 min. Marking patterns on days 0, 1, and 7 were assessed under UV trans-illumination, digitally photographed, and the number and area of marks scored by two observers (S.A.W. and G.M.V.) blind to the treatment of the animal. Inter-rater reliability for this test was >0.9.

### Statistical Analyses

#### Body weight

The body weight data were analyzed as a split plot in space and time [54]. The linear statistical model contained the fixed effects of diet, sex, day and all possible interactions with diet, sex and day. To determine whether there were litter effects, source (dam x male) within diet was used as the denominator of F for diet, source within d x sex was used as the denominator for sex and interaction of diet x sex, source within d was used as the denominator of F for d and the remaining interaction used the residual mean square as the denominator of F. Mean differences in body weight were determined by using Fisher’s protected Least Significant Difference (LSD).

#### Barnes maze data analyses

Continuous random variables assessed in the Barnes maze, including latency and error rate, were analyzed as a split plot in space and time [54]. The linear statistical model contained the fixed effects of diet, sex, day and all possible interactions with diet, sex and day. To determine whether there were litter effects, source (dam x male) within diet was used as the denominator of F for diet, source within day x sex was used as the denominator for sex and interaction of diet x sex, source within d was used as the denominator of F for day and the remaining interaction used the residual mean square as the denominator of F. Mean differences in body weight were determined by using Fisher’s protected Least Significant Difference (LSD).

The search strategy data were analyzed by using a repeated measurement design with PROC GLIMMIX and SAS version 9.2 software analyses (SAS Institute, Cary, NC). This analysis used a cumulative logit link and a multinomial distribution, i.e. all three search strategies were included in this analysis. Since this initial analysis indicated a significant three-way interaction between diet x sex x day, another cumulative logit analysis for each d was performed, where diet, sex, and diet x sex interaction were modeled. To pinpoint the differences further, a third analysis on search strategy was performed on which the two less efficient strategies (1 and 2) were combined and compared against the more efficient search strategy (3), thereby resulting in a binomial distribution. The PROC GLIMMIX was again used. Here the model contrasted diet, sex, diet x sex effects for each day with a logit link. The differences between the least square means were based on average logits. Tabled data were converted to probabilities, which is the probability of the treatment group employing one of the less efficient search strategies compared to the most efficient and direct search strategy.

#### Elevated plus maze (EPM) data analyses

The proportion of total time spent in open and closed arms and immobile, as well as total number of arm entries, average velocity, and total distance travelled were analyzed by a split plot design, as described above. The main variables included the effects of sex, diet, and sex x diet.

#### Territorial marking analyses

The data were analyzed by a repeated measurement by split plot, and time [55]. The individual ID within maternal diet was used as the denominator of F to test the effects of maternal diet on territorial marking. The residual means square was used as the denominator of F for testing day and interaction of maternal diet x day. Additionally, as two individuals (S.A.W. and G.M.V.) determined the number of urinary marks, the fixed effects in the model were assessed according to the person doing the assessments, diet, and all possible interactions. Animal ID within “person assessing data” and day was used as the denominator of F for the person assessing and maternal diet and interaction of these two variables. Residual means square was used as the denominator of F for testing day and all possible interactions of day with person assessing data and diet [55].

## Results

### Effect of Developmental Exposure to EDC on Body Weight

**Figure 1:**
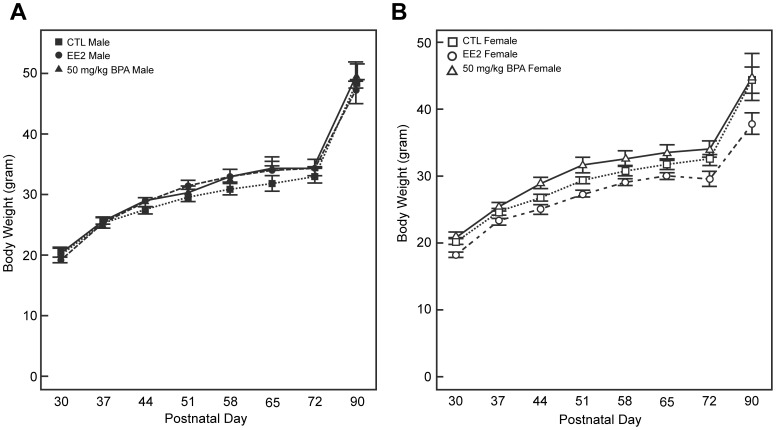
Body weight measurements of California mice males and females exposed to BPA, EE, and CTL diet. Measurements of body weight from 30 to 90 ds of age indicated that in males developmental exposure to BPA or EE did not affect body weight gain. In contrast, females exposed to EE weighed less than CTL females, EE males, and BPA females (P<0.05).

Offspring weight was tracked from 30 to 90 days of age to determine whether developmental exposure to BPA or EE effects during early development altered subsequent body weight gain and whether it occurred in a sex dependent manner. Data analysis revealed that there was a sex x maternal diet interaction (P<0.0003), but there was no three way interaction between sex x maternal diet x week (P = 0.9969). Further comparison of the overall average F1 female body weight revealed that those exposed during development to EE weighed less than their CTL and BPA counterparts (27.6±1.02, 30.5±0.8, and 31.5±1.0 grams, respectively; P values ranging from 0.03 to 0.008) (Figure 1). No significant differences were evident in the average male offspring body weight, although there was a trend for the BPA-exposed males to weight more than the CTL males (32.5±1.0 *versus* 30.1±0.9, P = 0.07).

### Spatial Learning

**Figure 2:**
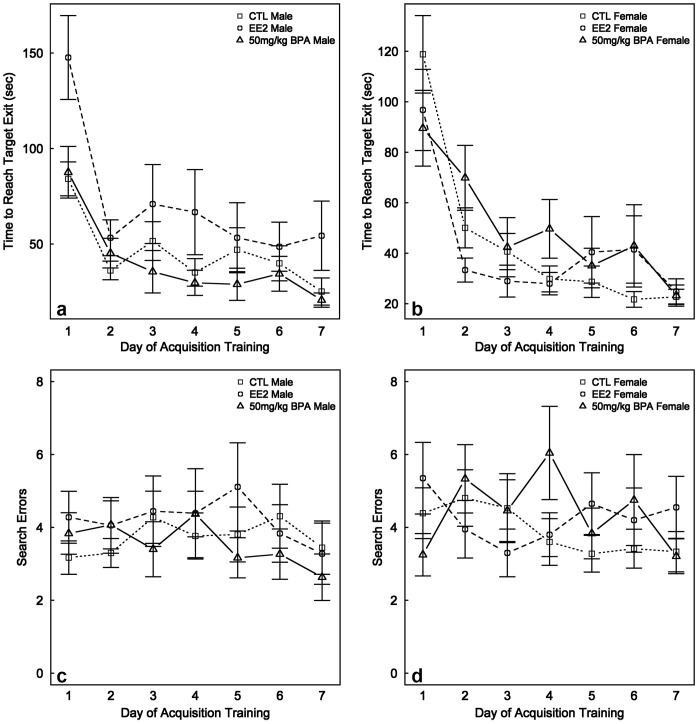
Measurements of latency (a and b) and error rate (c and d) in the Barnes Maze for California mice males and females. In males, developmental exposure to BPA or EE did not affect latency or error rate in the Barnes Maze compared to CTL males. Likewise, no effects were observed in BPA- or EE-exposed females compared to CTL females.

The Barnes Maze was used to test whether developmental exposure to BPA or EE affected spatial navigation. In contrast to our previous results with deer mice [35], [44], latency in this maze, i.e. time to reach the escape hole, was not affected by either maternal diet or offspring sex (P = 0.7 to 0.8) (Figure 2). When the results for all maternal diets and both sexes were combined, there was a decrease in latency from d 1 to d 7 (102.4±5.1 sec to 26.6±5.1 sec; P<0.0001). However, error rate was not affected by maternal diet, sex, or days of testing (P = 0.3 to 0.5) (Figure 2).

**Figure 3:**
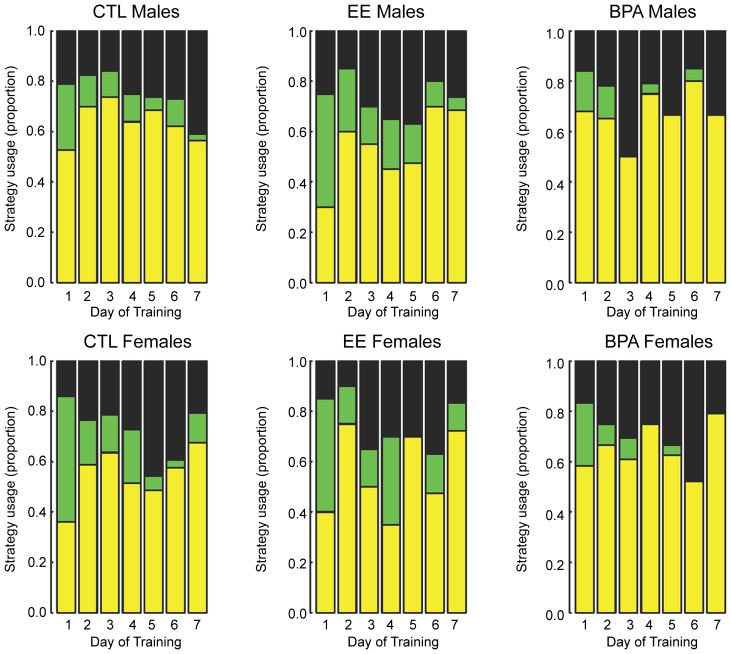
Barnes maze search strategy. The graph depicts the usage of random (yellow), serial (green), and direct (black) search strategies by CTL, EE-, and BPA-exposed males and females. During the seven d trial period, there were no consistent differences in search strategy based on maternal diet or sex.

Multinomial analysis of all three search strategies revealed that on d 4, there was a difference based on maternal diet but not sex (P = 0.04 and P = 0.7, respectively). On d 6, however, there was a significant difference based on offspring sex but not maternal diet (P = 0.03 and P = 0.5, respectively), as described below. However, the multinomial analysis that compares all three search strategies against each other is not sufficient to determine the specific nature of these changes. In essence, this analysis only indicates an overall trend based on maternal diet or sex; whereas the binomial analysis provides more definitive assessments to be made. No other differences were evident on any other trial days based on multinomial analysis. When the less efficient search strategies (random and serial) were combined and compared against the more efficient direct search strategy with a binomial analysis, the only difference based on maternal diet and sex occurred on d 6 when the males were more likely to use one of the more inefficient search strategies than the females (79.7% *versus* 59.0%, P = 0.02) (Figure 3). However, on no trial day did either sex convert to using the more efficient search strategy in preference to less efficient search strategies.

### EPM

**Figure 4:**
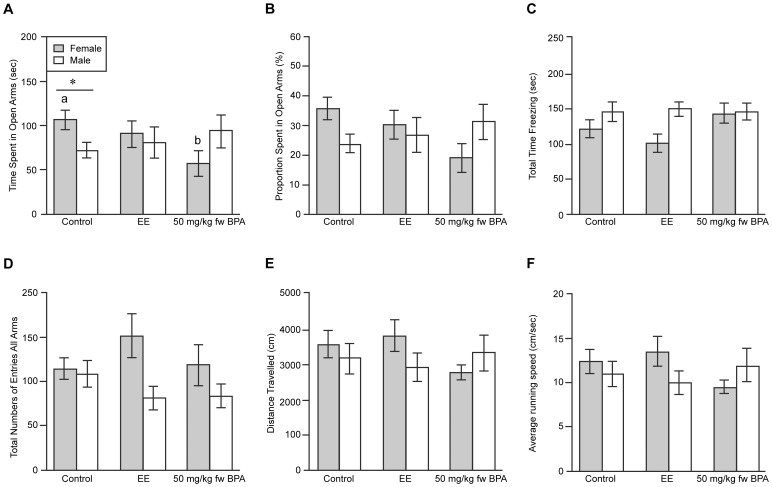
Measurements of exploratory and anxiety-like behaviors in EPM. a) Time spent in the open arms of the EPM. No difference was evident in time spent in the open arms between BPA- and EE-exposed males compared to CTL California mice males. In contrast, CTL female California mice spent more time in the open arms than BPA-exposed females and CTL males. ^a,b^Differences between maternal diets within sex (P<0.05), and ^*^differences between sexes within maternal diet (P = 0.05). b) Proportion of time spent in open arms, c) Time spent immobile, d) Total number of entries in all arms, e) Distance travelled, and f) Velocity. None of these other measurements were affected by sex or maternal diet.

In contrast to our previous results with polygynous deer mice [35], [36], no differences were observed for any of the parameters, including time spent in open and closed arms, time spent immobile, and distance travelled for males developmentally exposed to BPA or EE when they were compared to CTL males (Figure 4). However, there was significant interaction between maternal diet and sex for time spent in the open arms (P<0.04). This significant interaction occurred because CTL females spent more time in the open arms than BPA-exposed females (108.3±12.9 versus 55.0±16.2 seconds, P = 0.03), suggesting that BPA exposure during development had led to increased anxiety-like behaviors, with accompanying reduced exploration of the maze. Additionally, CTL female mice spent more time in the open arms than CTL males (108.3±12.9±72.4±13.3 seconds, P = 0.05). No other parameters for the EPM testing, including time spent immobile, total number of entries in all arms, distance travelled, and velocity, differed based on sex and maternal diet (Figure 4).

### Territorial Marking

**Figure 5:**
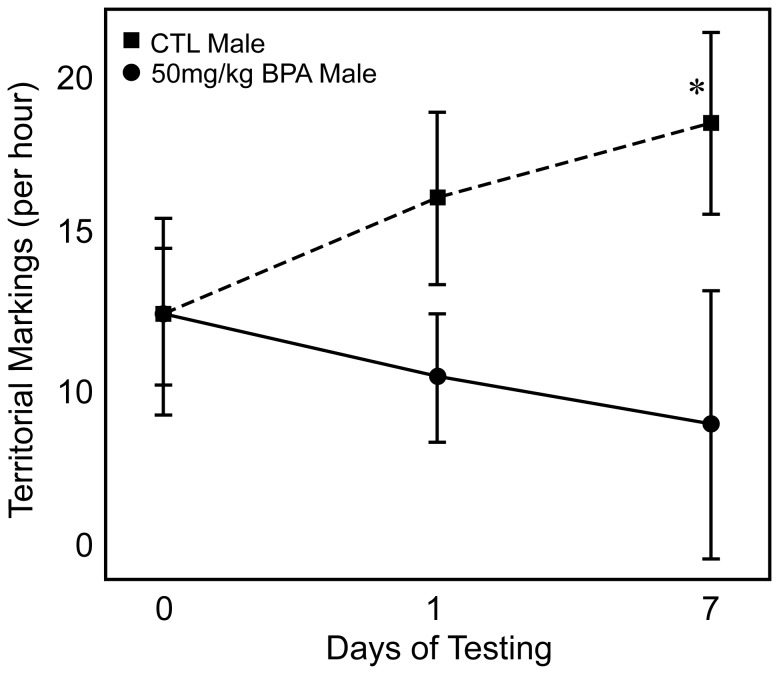
Measurement of territorial marking in BPA-exposed California mice males compared to CTL males. There was no difference on d 0 between BPA-exposed and CTL males, who did not have any visual or other sensory contact. When BPA-exposed and CTL males were placed in the same cage with a barrier between them, BPA-exposed males exhibited a trend to engage in less territorial marking on the first d of exposure (P<0.07) and even more so seven ds later than CTL males (*, P<0.003).

California mice use scent or territorial marking to establish their home range [40]–[42]. Therefore, we sought to determine if exposure to BPA disrupted this behavior. When males were housed individually to obtain basal marks (d 0), BPA-exposed males and CTL males did not differ in the number of urine marks they created (12.4±1.5 *versus* 12.4±2.1, P = 0.5) (Figure 5). By contrast, when the exposed and CTL males were co-housed in the same cage that had been separated into compartments by a propylene clear wall to permit visualization but not direct physical interaction, as early as d 1, the BPA-exposed males tended to engage in less urinary marking than CTL males (10.5±1.4 *versus* 16.2±1.8, P = 0.07) (Figure 5). This difference between the exposed males and CTL males persisted and became more pronounced when the same animals were re-tested seven days later (9.0±2.9 *versus* 18.6±2.0, P = 0.003) (Figure 5). CTL males increased their urinary marking on d 7 when they were in the presence of BPA-exposed male compared to their basal marking pattern on d 0 when the animals were housed alone (P = 0.03). In contrast, the BPA-exposed males did not demonstrate any increase in territorial marking on d 7 in the presence of another male compared to d 0, when they were singly housed (P = 0.2).

## Discussion

As spatial learning may not be under strong sexual selection in the California mice [44], we predicted that developmental BPA exposure would not lead to a sex-dependent disruption of spatial learning in this species. On the other hand, our expectation was that other sexually selected traits associated with the monogamous life history of the California mouse would be affected by BPA exposure. In the Barnes Maze, BPA-exposed males exhibited comparable latencies, error rates, and search strategies as CTL males, i.e. their performance was not compromised by BPA exposure. Neither CTL nor BPA-exposed male California mice shifted from a random/serial search strategy to a more direct search strategy during acquisition training. Instead, males generally engaged in random and serial search strategies to locate the escape hole, although latency did decrease significantly over the 7 days of training across all three diet groups and in both sexes, i.e. they found the hole much faster at the end than they did at the beginning of the trial.

Developmental exposure to BPA and EE failed to influence the behavior of males in the EPM, again providing a contrast with the earlier deer mouse study, in which males showed higher anxiety-like behavior following developmental BPA exposure [35]. However, one unexpected feature of the current study with the California mouse was that CTL females exhibited greater exploratory behavior in the EPM, than either CTL males or BPA-exposed females (Figure 4). In other words, BPA exposure reduced female exploratory behavior to the level of male California mice, again supporting the hypothesis that BPA would exhibit sex-by-species effects. On the other hand, there was no difference in exploratory behavior between EE-exposed females and CTL females, suggesting that the effects of BPA in California mice females are estrogen receptor independent. Whether California mice females in the wild are more likely to forage or rely on increased exploration than their male partners is unclear from the existing literature, but comparable laboratory findings have been reported for female Mongolian gerbils (*Meriones unguiculatus*), acutely exposed to BPA after being paired to a male [56]. Another study with laboratory mice (*Mus musculus*) also demonstrated that developmental exposure to BPA abolished sex differences in exploration and emotional responses [24]. The lack of effect of EE exposure on the exploratory behaviors of California females in the EPM is also intriguing in that it emphasizes that BPA is not acting simply as an estrogen analog.

Consistent with our hypothesis that sexually selected traits would be particularly susceptible to EDC, was the effect on male marking behavior. Androgens appear to be required for the territorial marking response of monogamous Mongolian gerbils [57]–[60] and a variety of other species [61]–[63], including laboratory mice [64]. Unlike the latter [65], where several EDC altered basal marking, in California mice basal territorial marking was not affected in BPA-exposed males. Instead, the normal surge in territorial marking by BPA-exposed males was suppressed only when a CTL male was visible in the testing arena (Figure 5). Possibly, control males achieve dominant status over to BPA exposed males during the familiarization, co-housing period (d1-6), and, as a consequence, deposit more urine marks on day 7, as has been been shown with a similar study design with laboratory mice [53]. While not directly quantified, our preliminary assessments indicated that when the males were co-housed between days 1–6, there were some differences in aggressive behaviors. However, no follow-up studies were performed to explore this phenomenon further due to animal welfare concerns. Nevertheless, reduced territorial marking and presumed low dominance in the wild could have reproductive consequences for male California mice. One possibility is that BPA exposed males will exhibit lower mate guarding and, as a consequence, increase their risk of cuckoldry [66], but this hypothesis remains to be tested. We are currently exploring the effects of BPA exposure on male behaviors when partnered with females and especially the biparental responses during the immediate post-partum period, where the males contribute significantly to the care of the pups [38], [67]–[71]. Reduced parental input by either parent as a result of their early exposure to BPA may have a negative input on the rearing of their pups. It is possible, for example, that poor parenting by the father as a result of his BPA exposure leads to harmful consequences for his offspring even though neither the mother nor her gametes have ever experienced BPA exposure.

In conclusion, the current studies are consistent with the hypothesis that sexually selected traits are unusually susceptible to endocrine disruption. In cases where there is no evidence for sexual selection, such as learning to navigate efficiently through the use of intramaze visual cues in the Barnes maze or exploratory behavior in an EPM, California males, unlike deer mice males [35], [36], are not affected by developmental exposure to either EE or BPA, whereas urinary marking behavior when confronting an unfamiliar male was suppressed. On the other hand, unexposed females engaged in more exploratory behavior than males of the species, and this aspect of female behavior was compromised by BPA but not EE exposure. Together, the experiments confirm that in assessing the effects of environmental chemicals on behavioral traits, the species, its sex and the targeted behavior must be selected carefully. Moreover, any study that seeks to examine the impact of developmental exposure to endocrine disruptors on behavioral outcomes in humans may need to consider traits where measurable differences exist between the sexes [72]–[74].
